# Antileishmanial activity of sulphonamide nanoemulsions targeting the **β**-carbonic anhydrase from *Leishmania* species

**DOI:** 10.1080/14756366.2018.1463221

**Published:** 2018-04-30

**Authors:** Verônica da Silva Cardoso, Alane Beatriz Vermelho, Eduardo Ricci Junior, Igor Almeida Rodrigues, Ana Maria Mazotto, Claudiu T. Supuran

**Affiliations:** a Bioinovar-Biotecnologia: Unidade de Biocatálise, Bioprodutos e Bioenergia (BIOINOVAR), Instituto de Microbiologia Paulo de Góes, Universidade Federal do Rio de Janeiro, Rio de Janeiro, Brazil;; b Departamento de Medicamentos, Laboratório de Desenvolvimento Galênico (LADEG), Universidade Federal do Rio de Janeiro, Rio de Janeiro, Brazil;; c Departamento de Produtos Naturais e Alimentos, Faculdade de Farmácia, Laboratório de Bioprospecção de Antimicrobianos Naturais (LABAN), Universidade Federal do Rio de Janeiro, Rio de Janeiro, Brazil;; d NEUROFARBA Department, Università degli Studi di Firenze, Sezione di Scienze Farmaceutiche, Florence, Italy

**Keywords:** Sulphonamide, *Leishmania*, nanoemulsions, β-carbonic anhydrase

## Abstract

The β-carbonic anhydrase (CA, EC 4.2.1.1) from *Leishmania* spp. (LdcCA) is effectively inhibited by aromatic/heterocyclic sulphonamides, in the low nanomolar range, but no *in vitro* antileishmanial activity was detected for such compounds. We formulated some of these sulphonamides as nanoemulsions (NEs) in clove oil, and tested them *in vitro* against *Leishmania infantum* MHOM/BR/1974/PP75 and *Leishmania amazonensis* IFLA/BR/1967/PH8 strains. Interesting inhibitory concentrations IC_50_ were observed for some of the sulphonamides NEs, with IC_50_ as low as 3.90 µM (**NE-3F**) and 2.24 µM (**NE-5B**) for *L. amazonensis* and 3.47 µM (**NE-5B**) for *L. infantum.* Some of the investigated NEs displayed toxicity for macrophages beyond the parasites. For the same nonoemulsions, a selective index (SI) greater than for Amphotericin B. Haemolytic assay using human red blood cells indicate that the NEs were less cytotoxic than amphotericin B, a widely used antifungal agent. NEs demonstrated to be an excellent strategy for increasing the penetration of these hydrophilic drugs through membranes, with a huge increase of efficacy over the sulphonamide CA inhibitor (CAI) alone.

## Introduction

Carbonic anhydrases (CA, EC 4.2.1.1) are widespread enzymes in organisms all over the phylogenetic tree^1–5^. CAs are metalloenzymes that catalyses the reversible hydration of CO_2_ to bicarbonate with a proton release^6^. They are grouped in seven distinct families, named α-, β-, γ-, δ-, ζ-, η-, and θ-Cas, classified according the sequence similarity/divergence^3^. Due the importance of CAs in cell physiology, their inhibitors possess a range of pharmacologic applications in various fields, such as for antiglaucoma drugs^7^, diuretics^8^, antiepileptics^9^, antiobesity agents^10^, as well as antitumor agents/diagnostics^11^. Recently, the potential use of CA inhibitors (CAIs) as anti-infective started to be considered for obtaining antibacterials^12–14^, antifungals^15^^,^^16^, and antiprotozoan agents^17–19^, with a novel mechanism of action, in the search of agents devoid the resistance problems common to most classes of clinically used such drugs^20^.

Leishmaniasis is a parasitic infectious disease caused by several species of *Leishmania,* an obligate intracellular protozoan parasite of humans that resides and multiplies in macrophages^21^. It is associated with significant rates of morbidity and mortality in many countries around the world. Leishmaniasis presents three main different clinical forms, visceral, cutaneous, and mucocutaneous^22^. There is no effective vaccine to prevent human leishmaniasis and the drugs available to chemotherapy have several limitations, as side effects and resistance to classical chemotherapy^21^^,^^23^. Thus, the search for new drug targets is required to develop newer therapies, and CAs are a promising target. The species used in this work, *Leishmania* (*L.*) *amazonensis* and *Leishmania* (*L*.) *infantum causes visceral Leismaniosis.*

We have reported that *Trypanosoma cruzi*, the aetiological agent of Chagas diseases^18^, another parasitic protozoan, encodes for an α-CA, called TcCA^24^, which was inhibited *in vitro* by many sulphonamide CA inhibitors (CAIs), in the low nanomolar or subnanomolar range^24^^,^^25^. However, *in vivo*, the growth of the parasite was not inhibited by such sulphonamides^24–28^. Other protozoa, such as *Plasmodium falciparum*, encode for CAs belonging to the η-CA class^18^, whereas *Leishmania* spp. possess β-CAs^29^. In earlier works from our groups we have shown that sulphonamides and thiols, well-known classes of CAIs, effectively inhibit *in vitro* this enzyme (called LdcCA as it has been cloned from the genome of *Leishmania donovani chagasi*). The sulphonamides showed inhibition constants varying between 50.2 nM and 9.25 µM, whereas some heterocyclic thiols inhibited the enzyme with K_I_s in the range of 13.4–152 nM^29^. Some of these thiols were shown to efficiently inhibit the *in vivo* growth of *Leishmania chagasi* and *L. amazonensis* promastigotes, by impairing the flagellar pocket and movement of the parasites and causing their death, whereas the sulphonamides, some of which showed similar inhibitory power *in vitro* as the thiols, were devoid of any such *in vivo* effects^31^. We hypothesised that these differences between the two classes of CAIs are due to the very polar nature of the sulphonamides, which interferes with their penetration through biological membranes of the pathogens in order to inhibit the enzyme, responsible for the pH regulation and probably other physiologic effects^30^. This is the reason why we investigated the possibility to enhance the bioavailability of the sulphonamide CAIs, by formulating them as nanoemulsions (NEs) in clove oil^30^^,^^31^.

The majority of NEs are dispersions of oil droplets in water with diametre between 20 and 200 nm. A recent study with sulphonamide NEs and *Trypanosomas cruzi* demonstrated that this represents indeed a good strategy to enhance the penetration of the drugs in the parasites^32^. Most NEs present small droplet size that allows the Brownian motion of the drops retarding their sedimentation or coalescence. Thus, NEs present kinetic stability^30^, promoting tissue permeation and penetration of drugs. Their nanometric droplets have large relative surface area, facilitating the contact of the nano-carrier with the biological membranes or tissues, and consequently favouring drug permeation and retention. In this paper, we present the antileishmanial activity of sulphonamide CAIs formulated as NEs in clove oil.

## Materials and methods

### Materials

Clove oil (*Eugenia caryophyllus*) was purchased from Ferquima Ltd. (Sao Paulo, Brazil). Pluronic F-127, a nonionic block-copolymer surfactant of (poly(ethylene oxide)-block-poly(propylene oxide)-block-poly(ethylene oxide)) (EO_100_PO_66_EO_100_), with MW 12,600, and HLB 22, was purchased from Sigma-Aldrich (St. Louis, MO). Dulbecco’s modified Eagle’s medium (DMEM), resazurin, amphotericin B, and thiazolyl blue tetrazolium bromide (MTT) were purchased from Sigma-Aldrich (St. Louis, MO). Fetal bovine serum (FBS) was purchased from LGC Biotecnologia (São José, Brazil).

### Chemistry

**Figure 1.**
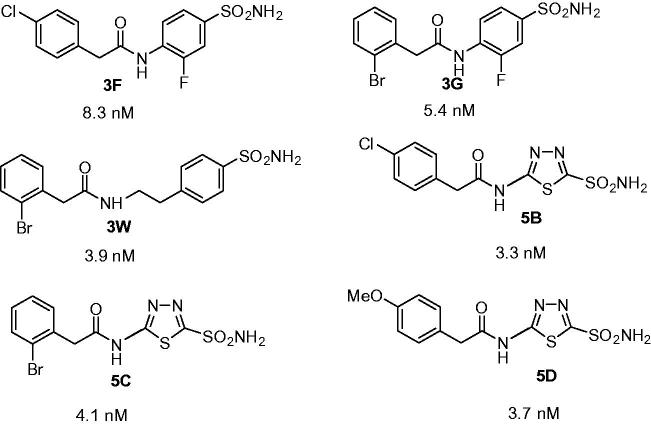
Sulphonamides **3F, 3G, 3W, 5B, 5C** and **5D** used in the study and their LdcCA inhibitory action (K_I_s, nM).

Sulphonamides **3F, 3G, 3W, 5B, 5C** and **5D** (Figure 1) used in the experiments were reported in an earlier work from our groups^25^, but they were not tested earlier as LdcCA inhibitors.

### CA activity measurements and inhibition studies

An Applied Photophysics (Oxford, UK) stopped-flow instrument has been used for assaying the CA catalysed CO_2_ hydration activity^33^. Phenol red (at a concentration of 0.2 mM) has been used as indicator, working at the absorbance maximum of 557 nm, with 20 mM Tris (pH 8.4) as buffer, and 20 mM Na_2_SO_4_ (for maintaining constant the ionic strength), following the initial rates of the CA-catalysed CO_2_ hydration reaction for a period of 10–100 s^33^. The CO_2_ concentrations ranged from 1.7 to 17 mM for the determination of the kinetic parameters and inhibition constants. For each inhibitor at least six traces of the initial 5–10% of the reaction have been used for determining the initial velocity. The uncatalysed rates were determined in the same manner and subtracted from the total observed rates. Stock solutions of inhibitors (10 mM) were prepared in distilled-deionised water and dilutions up to 0.01 nM were done thereafter with the assay buffer. Inhibitor and enzyme solutions were preincubated together for 15 min at room temperature prior to assay, in order to allow for the formation of the E-I complex. The inhibition constants were obtained by non-linear least-squares methods using PRISM 3, as reported earlier^34–36^, and represent the mean from at least three different determinations. All CA isoforms were recombinant ones obtained in-house as reported earlier^34–36^.

### Nanoemulsion preparation

The oil-in-water (O/W) NEs were prepared by high-energy method using an ultrasound processor (Hielscher model UP100H, Hielscher GmbH, Berlin, Germany), according to the method descripted by Senna et al.^31^. Oil phase was prepared by sulphonamides dissolution in the clove oil. An amount of 5 mg of drug was weighted in a microtube and 1 ml of clove oil was added. The tube was agitated for 1 min for obtaining of the drug solution (5 mg/ml). Aqueous phase was prepared by adding 1 g of Pluronic F127^®^ in 8 g of water. Then 1 ml of oil phase (drug dissolved in clove oil) was added to 9 ml of aqueous phase (Pluronic F127 in water) under constant ultrasound homogenisation (amplitude 80%, continuous cycle no. 1) during 5 min in an ice bath at 5 °C to prevent heating of the dispersion. A transparent NE was obtained at a concentration of 500 µg/ml.

### Determination of droplet size

Determination of droplet size and polydispersity index (PDI) were measured, using the dynamic light scattering (DLS) method with a Malvern model 90S NanoSizer^®^ (London, UK). NEs were diluted in distilled water at 1:10 and analysed in a cell with 1 cm optical path at room temperature (25 °C). These analyses were conducted in three runs with 15 readings. The values shown are the mean ± standard deviation of three measurements for each formulation. The PDI reflects the sample quality in the parameter homogeneity of the droplet diameter. PDI results lower than 0.3 were considered satisfactory^37^.

### *Leishmania* cultures

*Leishmania (infantum* MHOM/BR/1974/PP75 and *L. amazonensis* IFLA/BR/1967/PH8 were donated by the Leishmania Type Culture Collection (LTCC) of Oswaldo Cruz Institute/Fiocruz (Rio de Janeiro, Brazil). Parasite cultures were maintained in PBHIL medium supplemented with 10% of FBS at 26 °C^38^.

### RAW 264.7 macrophage cell line culture

RAW 264.7 macrophages were obtained from the National Institute of Metrology, Quality and Technology (Inmetro, Rio de Janeiro, Brazil) and maintained in DMEM medium supplemented with 10% FBS at 37 °C in a 5% controlled CO_2_ atmosphere. Cell maintenance was performed every 48–72 h, time necessary for cells to achieve confluent monolayers.

### Antileishmanial assay

The antileishmanial activity of the sulphonamide NEs was evaluated by the microdilution technique. First, polystyrene 96-well plates were used to serially dilute the samples in a 10% FBS-supplemented PBHIL medium. Amphotericin B and NEs prepared without the sulphonamides were used as positive and negative controls, respectively. *L. amazonensis* and *L*. *infantum* promastigote forms were harvest at late log phase of growth (96 h), washed twice with PBS and resuspended in fresh culture medium to a final concentration of 5 × 10^6^ parasites/ml. Then, 100 µl of each parasite suspensions were added to the plates, and the samples were adjusted to final concentrations ranging from 1 to 128 µM. After 120 h incubation period at 26 °C, parasites viability was assessed by adding 50 µL of resazurin solution (0.005%) as previously described by Rolon et al.^39^. The minimal inhibitory concentration (MIC) was determined as the lowest concentration capable of inhibiting *in vitro* growth of the parasites. The 50 and 90% inhibitory concentrations (IC_50_ and IC_90_) were calculated by regression analysis using Microsoft Excel 2013 software.

### Cytotoxic assay

Sulphonamide NEs cytotoxicity was performed using tetrazolium dye MTT colorimetric assay. RAW 264.7 macrophages were harvest after confluent monolayer achievement. The cells were washed twice with PBS and a cellular suspension of 10^6^ cells/ml was prepared in fresh DMEM culture medium. Aliquots of 100 µl of the cellular suspension were placed into polystyrene 96-well plates, and then incubated at 37 °C in a 5% CO_2_ atmosphere for 6 h to allow for adherence of macrophages. After this period, the adherent cells were subjected to treatment with several concentrations of the sulphonamide NEs (1–128 µM), and then incubated for additional 48 h. Finally, 20 µl of a MTT solution (5 mg/ml) were added to each well and the plates incubated for 4 h as previously described^40^. Macrophage viability was determined after formazan crystals solubilisation with DMSO followed by the absorbance measurement at 570 nm using a SpectraMax M5 spectrophotometer (Molecular Devices, Los Angeles, CA). The 50% cytotoxic concentrations were calculated by regression analysis using Microsoft Excel 2013 software.

### Selective index determination

The selective index (SI) for promastigote forms of *L. amazonensis* and *L. infantum* was calculated by the ratio between the CC_50_ for RAW 264.7 macrophages and the IC_50_ for the parasites. Samples with SI values >10 were considered as low cytotoxic agents^41^.

### Haemolytic assay

Haemolytic activity was evaluated as described previously by Ishnava and Shah with a slight modification^42^. Human erythrocytes from healthy individuals were collected in vacuum tubes containing EDTA as anti-coagulant. The erythrocytes were harvested by centrifugation for 10 m at 2500 rpm at 20 °C, and washed three times in PBS. To the pellet, PBS was added to yield a 10% (v/v) erythrocytes/PBS suspension. The 10% suspension was then diluted 1:10 in PBS. Aliquots of 100 µl of erythrocytes suspension was added, in triplicate, to 100 µl of a two-fold dilution series of sulphonamides NEs and amphotericin B (at concentrations of 128, 64, 32, 16, 8, 4, 2, and 1 µM, respectively, in the same buffer) in microtubes. Negative and positive controls were performed by replacing drug dilution with PBS or with 200 µl of % Triton X-100 for the total haemolysis, respectively. The tubes were incubated for 1 h at 37 °C and then centrifuged for 10 min at 2000 rpm at 20 °C. From the supernatant fluid, 150 µl was transferred to a flat-bottomed microtiter plate, and the absorbance was measured spectrophotometrically at 540 nm. Five concentrations are required for HC_50_ calculation. The following formula was used to find out the percentage of haemolytic activity: [A540 treated sample − A540 of buffer)/(A540 of Triton X-100 − A540 of buffer)] × 100.

### Statistical analysis

The data of the experiments are being carried out through the programme Prism 5.01 GraphPad (GraphPad Software, La Jolla, CA), being considered values statistically significant those with values of the standard deviation (SD), *p* < .05.

## Results and discussion

### Carbonic anhydrase inhibition with sulphonamide 3F, 3G, 3W, 5B, 5C, and 5D and preparation of their clove oil NEs

**Figure 2.**
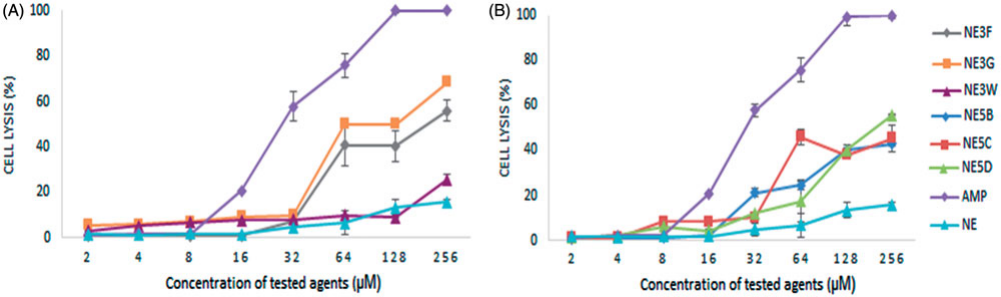
Haemolysis caused by different agents: amphotericin B (AMP) and nanoemulsions containing inhibitors. (A) **NE-3F, NE-3G, NE-3W**, AMP, and NEs; B. **NE-5B, NE-5C, NE-5D**, AMP, and NEs. (B) Haemolysis was determined by an absorbance reading at 540 nm and compared to haemolysis achieved with 1% Triton X-100 (reference for 100% haemolysis). Values are expressed as mean ± standard deviation (*n* = 3).

Sulphonamides **3F, 3G, 3W, 5B, 5C**, and **5D** (Figure 1) were investigated as *in vitro* inhibitors of LdcCA, the β-CA cloned and characterised earlier by our groups^29^. As seen from Figure 1, they act as highly efficient LdcCA inhibitors *in vitro*, with inhibition constants ranging between 3.3 and 8.3 nM. All of them are very effective inhibitors of the protozoan enzyme, making structure-activity relationship difficult to delineate. However, as mentioned above, sulphonamides structurally related to the ones investigated here did not show *in vivo* anti-leishmanial effects^29^. This is why we formulated here sulphonamides **1–6** as NEs in clove oil (Figure 2).

Sulphonamides **3F, 3G, 3W, 5B, 5C,** and **5D** were easily dissolved in clove oil in the concentration of 5 mg/ml. The NEs were produced with 10% of oil phase. NEs were prepared also without the drug in order to evaluate the stability, droplet size and PDI. The NEs obtained were yellow and transparent suggesting that the system was homogeneous with small droplet size (Table 1). Phase separation and precipitation of the drug were not observed, and the NE was considered stable in the concentration of up to 500 µg/ml.

**Table 1.** NEs size and polydispersity index.

Formulation/drug	Drug (mg)	Oil clove (ml)	AP (ml)	Size (nm)	PDI	Stability
**NE**	–	1	9	31.54 ± 0.413	0.105 ± 0.012	Stable
**NE-3F**	5	1	9	60.12 ± 2.36	0.274 ± 0.033	Stable
**NE-3G**	5	1	9	100.63 ± 2.05	0.262 ± 0.008	Stable
**NE-3W**	5	1	9	97.34 ± 2.82	0.264 ± 0.15	Stable
**NE-5B**	5	1	9	44.83 ± 0.753	0.123 ± 0.078	Stable
**NE-5C**	5	1	9	53.99 ± 1.12	0.233 ± 0.003	Stable
**NE-5D**	5	1	9	35.09 ± 0.575	0.165 ± 0.019	Stable

Drug concentration 500 µg/mL.

Mean ± SD of *n* = 3 determinations.

AP: aqueous phase containing detergent (Pluronic F127) and water.

NEs without the drug presented an average size of 31.54 nm. The NEs containing the drug presented average sizes between 35 and 100 nm, depending on the drug. The lowest average size was exhibited by **NE-5D** with a diameter of 35.09 nm. **NE-3G** and **NE-3W** exhibited the larger average size values with diameters of 100.63 and 97.34 nm, respectively. These NEs presented PDI below 0.3; indicating that the size distribution is homogeneous and monomodal^37^. Thus, we conclude that the inclusion method of the drugs in NEs was adequate, producing nanostructured samples with drops below 100 nm and size distribution homogeneous and monomodal.

### Anti-*Leishmania infantum/amazonensis* activity *in vivo*

The effect of NEs containing the sulphonamides **3F, 3G, 3W, 5B, 5C,** and **5D** on *L. amazonensis* and *L. infantum* promastigotes viability was assessed *in vitro*. The obtained results are summarised in Tables 2 and 3. All NEs displayed *in vitro* antileishmanial activity with great variations in the IC_50_ values, which ranged from 3.47 to 51.7 µM for *L. infantum*. The IC_50_ values for *L. amazonensis* did not vary that much, ranging between 2.24 and 18.26 µM. The best IC_50_ against these parasites were presented by **5B-NE**, followed by **3F-NE** (IC_50_ 3.90 µM) for *L. amazonensis* and **NE-3G** (IC_50_ 10.72 µM) for *L. infantum*.

**Table 2.** Viability assay of *L. amazonensis* promastigotes, cytotoxicity test using RAW 267.4 macrophage cells, and determining the selectivity index of nanoemulsions.

	*L. amazonensis*						
Nanoemulsions	**3F**	**3G**	**3W**	**5B**	**5C**	**5D**	AMP^6^
MIC^1^	128	>128	128	32	>28	>28	4
IC_50_^2^ µM	3.90 ± 1.96^a^	12.01 ± 0.58^b^	10.55 ± 4.51^b^	2.24 ± 0.178^c^	12.41 ± 0.45^b^	18.26 ± 5.28^d^	0.61 ± 0.01^e^
IC_90_^3^ µM	105.58 ± 30.63^a^	n.d.	92.74 ± 38.23^b^	22.46 ± 6.80^c^	n.d.	n.d.	1.23 ± 0.23^d^
CC_50_^4^ µM	8.13 ± 1.1^a^	6.77 ± 1.7^b^	3.21 ± 0.55^c^	6.51 ± 1.11^b^	8.04 ± 1.33^a^	6.75 ± 0.98^b^	1.07 ± 1.49^d^
IS_50_^5^	2.06 ± 0.17^a^	0.48 ± 0.21^c^	0.34 ± 0.12^d^	2.12 ± 0.11^a^	0.64 ± 0.22^c^	0.37 ± 0.09^d^	1.78 ± 0.01^b^

The means compared by the Student–Newman–Keuls test.

n.d.: not determined.

^1^MIC: minimum inhibitory concentration.

^2^IC_50_: concentration which reduced 50% of the proliferation of *L. amazonensis* promastigote stage.

^3^IC_90_: concentration which reduced 90% of the proliferation of *L. amazonensis* promastigote stages.

^4^CC_50_: concentration in μg·ml^−1^ cytotoxic for 50% of RAW 264.7 cells.

^5^SI: selectivity index = CC_50_/IC_50_.

^6^AMP: amphotericin B.

^a,b,c,d,e^In the lines: averages with equal letters do not differ statistically (*p*>.05).

**Table 3.** Viability assay of *L. infantum* promastigotes, cytotoxicity test using RAW 267.4 macrophage cells, and determining the selectivity index of NEs.

	*L. infantum*						
Nanoemulsions	**3F**	**3G**	**3W**	**5B**	**5C**	**5D**	AMP^6^
MIC^1^	>128	>128	>128	64	>128	>128	4
IC_50_^2^ µM	12.00 ± 4.3^a^	10.72 ± 2.68^a^	12.46 ± 1.35^a^	3.47 ± 0.35^c^	14.58 ± 0.88^a^	51.70 ± 5.18^b^	0.67 ± 0.10^d^
IC_90_^3^ µM	n.d.	n.d.	n.d.	52.03 ± 8.01^a^	n.d.	n.d.	1.01 ± 0.03^b^
CC_50_^4^ µM	8.13 ± 1.19^a^	6.77 ± 1.07^b^	3.21 ± 0.55^c^	6.51 ± 1.11^b^	8.04 ± 1.33^a^	6.75 ± 0.98^b^	1.07 ± 1.49^d^
IS_50_^5^	0.66 ± 0.17^a^	0.77 ± 0.21^a^	0.34 ± 0.12^a^	2.01 ± 0.11^b^	0.87 ± 0.22^a^	0.11 ± 0.09^c^	1.59 ± 0.01^d^

The means compared by the Student–Newman–Keuls test.

n.d.: not determined.

^1^MIC: minimum inhibitory concentration.

^2^IC_50_: concentration which reduced 50% of the proliferation of *L. infantum* promastigote stage.

^3^IC_90_: concentration which reduced 90% of the proliferation of *L. infantum* promastigote stages.

^4^CC_50_: Concentration in μg·ml^−1^ cytotoxic for 50% of RAW 264.7 cells.

^5^SI: selectivity index = CC_50_/IC_50_.

^6^AMP: Amphotericin B.

^a,b,c,d^In the lines: averages with equal letters do not differ statistically (*p*>.05).

IC_90_ values are summarised in Tables 2 and 3. The inhibitors **NE-3F, NE-3W, NE-5B** showed IC_90_ of 105.58 ± 30.63, 92.74 ± 38.23, 22.46 ± 6.80 µM, respectively, for *L. amazonensis*, and the compound **NE-5B** induced an IC_90_ of 52.03 ± 8.01 µM for *L. infantum*. The inhibitors **NE-3F, NE-3W,** and **NE-5B** against *L. amazonensis* and **NE-5B** for *L. infantum* promastigotes were able to inhibit the growth of the parasites. After 48 h in culture medium, no cell growth of *L. amazonensis* was observed at the concentration of 128 µM of the following inhibitors **3F, 3W** in NEs. NEs containing 5B at the concentrations 32 and 64 µM inhibited completely *L. amazonensis* and *L. infantum,* respectively. These values correspond the MIC for both Leishmanias (Tables 2 and 3).

It was not possible to determine the MIC and IC_90_ for promastigotes treated with NEs **NE-3G, NE-5C,** and **NE-5D** for *L. amazonensis* and **NE-3F, NE-3G, NE-3W, NE-5C,** and **NE-5D** for *L. infantum*. Probably the MIC and IC_90_ for these NEs are above 128 µM (first concentration studied).

On the other hand, the worst compound was **NE-5D** for *L. amazonensis* and *L. infantum*. Only the **NE-5B** showed IS_50_ above two for both parasites. Except for compounds **NE-5B** and **NE-3F**, all the others NEs containing inhibitors displayed more toxicity for macrophage cell than for parasites (IS_50_ < 1) (Table 4).

**Table 4.** Haemolitic index of nanoemulsions **3F**, **3G**, **3W**, **5B**, **5C**, and **5D**.

	Haemolysis (%)							
µM	**3F**	**3G**	**3W**	**5B**	**5C**	**5D**	**ANF**	Nanoemulsion without drug
HC_50_	168.28 ± 14.15^a^	138.96 ± 32.97^a^	>256	>256	168.62 ± 18.45^a^	>256	27.93 ± 1.71^b^	>256
256	55.81 ± 14.50	68.51 ± 1.3	25.79 ± 2.0	43.04 ± 1.1	45.32 ± 16.13	55.7 ± 10.4	100.0 ± 0	15.75 ± 1.24
128	40.43 ± 16.73	49.9 ± 10.2	9.03 ± 0.06	40.0 ± 0.93	38.01 ± 1.48	40.22 ± 2.1	100.0 ± 0	13.37 ± 3.49
64	40.87 ± 9.36	49.86 ± 0.8	10.0 ± 0.01	24.37 ± 2.9	45.89 ± 9.25	17.35 ± 9.5	75.7 ± 5.24	6.71 ± 5.33
32	7.17 ± 0.30	10.0 ± 0.04	8.06 ± 0.04	21.17 ± 2.1	10.11 ± 0.18	12.15 ± 0.3	57.81 ± 6.7	4.75 ± 2.66
16	1.10 ± 0.17	9.25 ± 0.43	7.74 ± 0.43	2.11 ± 0.20	8.24 ± 0.42	4.29 ± 0.50	20.81 ± 0.5	1.68 ± 0.51
8	1.11 ± 0.24	7.32 ± 0.56	6.96 ± 0.56	1.22 ± 0.38	8.21 ± 0.36	6.27 ± 0.46	2.09 ± 0.15	1.81 ± 0.22
4	1.20 ± 0.34	6.04 ± 0.07	5.13 ± 0.20	1.04 ± 0.06	1.16 ± 0.28	2.02 ± 0.04	2.01 ± 0.02	1.35 ± 0.37
2	1.00 ± 0.04	5.72 ± 0.49	3.13 ± 0.07	0.95 ± 0.09	1.02 ± 0.04	1.12 ± 0.20	1.18 ± 0.31	1.74 ± 0.76

The means compared by the Student–Newman–Keuls test.

^a,b^In the lines: averages with equal letters do not differ statistically (*p* > .05).

In parallel, NE cytotoxicity assays were performed on 267.4 macrophage cells. At the concentrations tested, NEs were less cytotoxic than Amphotericin B which is a potent leishmanicide drug with anti-*Leishmania* effect demonstrated *in* vitro and *in vivo* studies against the promastigote and amastigote forms of the Leishmania parasite^43^. Typically, assays in promastigote forms of the parasite are always present in the initial screening of candidate compounds for leishmanicidal drugs, since they are simple and inexpensive tests^44^.

Previous work from our group showed that thiols were more effective than sulphonamides in the inhibition of LdcCA from *L. donovani chagasi,* another *Leishmania* associated with the visceral form of the disease^29^.

Several studies have been demonstrated that nano-emulsified carrier systems have potential to solve problems with poor water solubility and poor membrane permeability of some drugs^45^. Moreover, the toxicity of some drugs can be reduced when they are nano-emulsified with an appropriate carrier^46–48^. Gupta et al. formulated a nano-emulsified carrier system with copaiba oil to improve the antileishmanial activity and oral bioavailability of amphotericin B^46^. They produced their NE by mixing a surfactant (TPGS, d-α-tocopheryl polyethylene glycol 1000 succinate), a co-surfactant (phosphatidyl choline (PC)), the oil and the drug, to form an O/W emulsion with particle size of 127 ± 21 nm and PDI of 0.11 ± 0.02. The IC_50_ value of amphotericin nanoemulsified with copaiba oil and plain amphotericin B against *L. donovani* was 0.018 ± 0.004 and 0.214 ± 0.06 µg/ml, respectively, demonstrating a nearly 12-fold reduction in the IC_50_ value. They also reported the haemotoxicity reduction of amphotericin B from 69.8 to 18.2% when formulated in the NE. These results are corroborated by Santos et al. who also observed increase *in vivo* anti-leishmanial activity and reduction of *in vitro* haemotoxicity of amphotericin B^47^. However, they did not use natural oil in NE composition, as their NE was prepared with medium chain triglycerides (MCT), Tween 80, and cholesterol in the oily phase and glycerol and amphotericin B in the aqueous phase, forming particles with 134.8 nm of mean size^47^. In our study, the compounds that did not inhibit significantly *Leishmania* spp. in plain solution but started to inhibit the tested parasites with IC_50_ values ranging from 2.24 to 18.26 µM and from 3.47 to 51.7 µM for *L. amazonensis* and *L. infantum*, respectively, when formulated as NEs. The improvement of the anti-leishmanial activity through the production of simple NEs of these sulphonamide CAIs is thus remarkable, whereas its preparation and in the number of components required to formulate the NE are also simplified compared to previously reported procedures^46^^,^^47^.

### Haemolytic assay

Human red blood cells provide a handy tool for toxicity studies of the compounds, because they are readily available, their membrane properties are well known, and their lysis is easily monitored by measuring the release of haemoglobin. At the concentration of 256 and 128 µM, amphotericin B (conventional anti-leishmanial therapeutic agent) exerted 100% haemolysis. In comparison, the NE without drug caused only 15.75 and 13.37% haemolysis. Taken together, our results indicate that oil glove present in the NEs have significantly less cytotoxic effects than amphotericin B. In all sulphonamides NEs, at concentrations below 16 µM, a haemolysis of less than 10% was found. These results showed that NEs containing the CAIs are promising for therapeutic drug trials.

The incorporation of sulphonamides in clove oil NE allowed the drugs to meet their intracellular targets, and to perform their anti-leishmanial activity. These inhibitors do not penetrate into the parasite without the NEs (data not shown). These results suggest that **5B-NE** present potential activity against amastigotes, since the NE penetrated into macrophages. Other interesting point that could be studied is the synergistic action between clove oil and **5B** on the anti-leishmanial activity. Oil clove (*Syzygium aromaticum*) has been reported to have anti-leishmanial activity due to high eugenol concentration^48^. The values of IC_50_ described by Islamuddin et al. against promastigotes and intracellular amastigotes of *L. donovani* were 21 and 15.24 mg/ml, respectively^48^. The clove oil caused apoptosis in the *L. donovani* cell parasite. Although promoting the parasite growth inhibition, the NEs investigated here also reduced the cell viability of macrophages. The macrophages cytotoxicity might be associated principally to the action of sulphonamides, although many such drugs are used for decades without any evidence of such effects^7^. The oil clove cytotoxicity reported by Islamuddin et al. revealed that there was no toxicity of eugenol-rich oil of *S. aromaticum* on RAW264.7 cells, even at 200 mg/ml^48^. Many substances with potential therapeutic applications have been discarded in the past because they were not administrable in a bioavailable form^48^. This study opens the possibility to formulated NEs containing the CAIs in order to increase the bioavailability of hydrophilic drugs, such as the sulphonamides.

## Conclusions

The best IC_50_ against both parasites were obtained by **5B-NE**, followed by **3F-NE** (IC_50_ 3.90 µM) for *L. amazonensis* and 3G-NE (IC_50_ 10.72 µM) for *L. infantum* NEs have demonstrated potential as a novel vehicle for delivery hydrophophilic drugs such as sulphonamide CAIs improving the bioavailability of the drug and demonstrating a potential use in the treatment of leishmaniasis.
